# Olanzapine is superior to lamotrigine in the prevention of bipolar depression: a naturalistic observational study

**DOI:** 10.1186/1471-244X-14-145

**Published:** 2014-05-19

**Authors:** Pei-Yin Pan, Meei-Shyuan Lee, Miao-Chi Lo, En-Lin Yang, Chin-Bin Yeh

**Affiliations:** 1 Department of Psychiatry, Tri-Service General Hospital, National Defense Medical Center, No. 325, Cheng-Kung Road, Sec. 2, Nei-Hu District, Taipei 114, Taiwan, R.O.C; 2 School of Public Health, National Defense Medical Center, No.161, Min-quan E. Road, Sec. 6, Nei-Hu District, Taipei 114, Taiwan, R.O.C

**Keywords:** Olanzapine, Lamotrigine, Bipolar disorder, Maintenance treatment

## Abstract

**Background:**

Bipolar disorder is a highly recurrent disease and has great impact on the function of patients. Depressive symptoms consist of more than 50% of life time during the illness and may lead to self harm or suicidal behaviors. Little is known about the antidepressant effects of olanzapine, an atypical antipsychotic, as monotherapy despite its indication for preventing manic episodes. In contrast, lamotrigine, a mood stabilizer, has been proven to be effective in preventing depression in patients with bipolar disorder. However, no studies have compared the efficacy between lamotrigine and olanzapine in the maintenance treatment of bipolar disorder. This enriched naturalistic study was implemented to assess the effectiveness of olanzapine and lamotrigine as monotherapy in the prevention of recurrence of bipolar disorder.

**Methods:**

Patients with bipolar disorder in a euthymic state (Young’s Mania Rating Scale (YMRS) score <12, and 21-item Hamilton Depression Rating Scale (HAM-D) score <7) for at least two months, having already received either olanzapine or lamotrigine as the maintenance treatment were recruited. The patients maintained with olanzapine (n = 22) were applied to olanzapine group whereas those maintained with lamotrigine (n = 29) were applied to lamotrigine group. They were followed up for 12 months. Differences in the efficacy between olanzapine and lamotrigine in recurrence prevention were analyzed. The Kaplan-Meier method was used to generate time-to-recurrence curves, and differences between the two groups were compared using the log-rank test.

**Results:**

Olanzapine had a significantly lower recurrence rate of depressive episodes than lamotrigine (20.0% vs. 57.7%, χ^2^ = 6.62, p = .010). However, olanzapine and lamotrigine had similar mania (15.0% vs. 0%, χ^2^ = 4.17, p = .075, Fisher’s exact test) and any mood episode (35.0% vs. 57.7%, χ^2^ = 2.33, p = .127) recurrence rates. Olanzapine was significantly superior to lamotrigine in the time to recurrence of depressive episodes (χ^2^ = 4.55, df = 1, p = .033), but there was no difference in the time to recurrence of any mood episode (χ^2^ = 1.68, df = 1, p = .195).

**Conclusions:**

This prospective naturalistic study suggests that olanzapine is more effective than lamotrigine in the prevention of depressive episodes in patients with bipolar disorder. Future large-scale randomized studies are warranted to validate our results.

**Trial registration:**

ClinicalTrials.gov ID NCT01864551.

## Background

Bipolar disorder is a highly recurrent psychiatric disorder, and nearly half of the patients experience subsequent episodes of illness within one year after the first episode [1,2], and a recurrence rate of up to 90% in the following 4–5 years [1,3-5]. Depressive symptoms and episodes dominate the course of illness [6] and may lead to social and family dysfunction, repeated hospitalizations and even suicidal behaviour [7,8]. However, the patients with bipolar disorder are more likely to have the comorbidities of anxiety, substance use disorder [9] and personality disorder [10] which complicate the treatment and may be associated with a higher recurrence rate [11] and poor prognosis [12-15]. In addition, the different subtypes (bipolar I, II or rapid cycling) [16-18] of bipolar disorder or the gender of the patient [19,20] may lead to different responses to treatments. Therefore, acute and prophylactic pharmacological treatment for bipolar depression is challenging for clinicians as only a few agents have been demonstrated to be efficacious.

In recent years, monotherapy with atypical antipsychotics has been found to be effective in the maintenance treatment of bipolar disorder [21]; however, the efficacy of only a few have been validated for the prevention of bipolar depression [22-24]. Olanzapine, one of the drugs approved by the Food and Drug Administration (FDA) for maintenance treatment, has been reported to be beneficial in the prophylaxis of depressive episode in terms of delaying the time to relapse into depression compared to placebo [25]. Among the atypical antipsychotics used in treating the acute phase of mood episodes, olanzapine is ranked as one of the two most optimal treatments for its preferable efficacy and lower drop-out rate in patients with bipolar disorder [26] that may contribute to fewer residual symptoms and better adherence which have been correlated with a lower risk of recurrence to bipolar depression [11]. Olanzapine has also been found to be effective in decreasing concurrent anxiety symptoms in patients with bipolar disorder [27,28] that are considered to be a predictor of depressive recurrence [11], and also to be associated with poor treatment response and worse symptom severity [29-31]. However, despite these potential advantages in the prophylaxis treatment for bipolar depression, few studies have compared olanzapine with other mood stabilizers in the prevention of depressive episodes in patients with bipolar disorder [32,33].

Lamotrigine is the most well-established mood stabilizer in the prevention of depressive recurrence in bipolar disorder [34], not only for its efficacy but also for its good tolerability and adherence [35,36]. Two large randomized control trials (RCTs) demonstrated that lamotrigine was superior to a placebo at lengthening the time to the recurrence of depressive episodes [37,38]. Nevertheless, there was no confirmed superiority of lamotrigine compared with other mood stabilizers such as lithium in the efficacy of prophylaxis treatment in bipolar depression [39]. In addition, no head-to-head trials of lamotrigine and atypical antipsychotics as monotherapy in the maintenance treatment of patients with bipolar disorder have been performed, although lamotrigine has been reported to show comparable efficacy with the combination of olanzapine and fluoxetine in terms of the incidence of relapse of bipolar depression [40].

The efficacy and potential detrimental impact of the long-term administration of prophylaxis treatment in the patients with bipolar disorder are controversial issues. Taking into consideration the emerging concern of the increased morbidity and mortality associated with atypical antipsychotics [41], mood stabilizers may be considered for maintenance treatment due to fewer adverse effects despite the modest therapeutic performance [36,42], although mortality after anticonvulsant treatment has been reported [43,44]. However, few studies have compared the effectiveness between atypical antipsychotics and mood stabilizers; and thus there is currently insufficient information to assess the balance between their advantages and acceptability in clinical practice. In view of the data from previous trials on olanzapine and lamotrigine, a parallel comparison of these two agents in the maintenance treatment of bipolar disorder is of substantial clinical applicability. Previous RCTs in this field were conducted with the limitation of highly selected patient populations and the restricted use of concurrent medications, and such designs do not correspond with an actual clinical setting. To investigate the effectiveness of olanzapine and lamotrigine in maintenance treatment, we conducted this open-label, enriched, naturalistic study with one year of follow-up in patients with bipolar disorder.

## Methods

### Patients

The participants, all screened at the psychiatric outpatient of the principal investigator’s affiliation which was a medical university hospital, were aged from 15 to 50 years and met the Diagnostic and Statistical Manual of Mental Disorders, fourth edition, text revision (DSM-IV-TR) criteria for bipolar disorder in the euthymic state (a Young’s Mania Rating Scale (YMRS) score <12, and a 21-item Hamilton Depression Rating Scale (HAM-D) score <7). Those who had already received either olanzapine or lamotrigine as the maintenance treatment for at least two months were enrolled in this study. Patients were excluded if they met the DSM-IV criteria for schizophrenia or schizoaffective disorder, or if they had severe medical diseases. Patients were also excluded if they had a history of rapid cycling (according to the DSM-IV-TR criteria) or were taking concomitant medications that might influence the metabolism of olanzapine or lamotrigine and those who were allergic to the two agents. All of the participants signed informed consent forms after the study had been thoroughly explained to them, and all received physical examinations and chart reviews regarding the laboratory measurements to confirm the medical history during outpatient visits. They were all evaluated by the principal investigator, a senior psychiatrist, for the severity of manic and depressive symptoms by the YMRS and HAM-D. The study was approved by the Institutional Review Board of Tri-Service General Hospital, National Defense Medical Center, Taipei, Taiwan.

### Study design and assessments

This study was an open, parallel, naturalistic observational investigation. The patients who received olanzapine as maintenance therapy before entering this study were defined as the olanzapine group, and those who received lamotrigine were defined as the lamotrigine group. The use of concomitant medications including antidepressants and benzodiazepines was allowed, but not other mood stabilizers or antipsychotics. The patients were followed up for 12 months and were scheduled to visit the outpatient clinic every four weeks. We defined symptomatic recurrence as a YMRS score ≥ 12 or a HAM-D score ≥ 7, or if the patient’s condition required an increase in the dosage of the original mood stabilizer or atypical antipsychotic. Syndromic recurrence was defined as when the patient’s condition met the DSM-IV-TR criteria for a manic or depressive episode, or if the patient had active suicide ideation. Adverse events were evaluated at each visit by asking the patients whether they have experienced any physical discomfort but no quantitative measurements were performed.

### Statistical analysis

All statistical analyses were performed with SPSS software version 17 (SPSS Inc., Chicago, IL, USA). To compare the baseline characteristics between the two groups, the chi-square test or Fisher’s exact test were used for categorical variables and the Student’s t test for continuous variables. The chi-square test and Fisher’s exact test were used to compare the recurrence rates of any mood episodes between the two groups at the end of follow-up. The Kaplan-Meier method was used to generate time-to-recurrence curves, and differences between the two groups were compared using the log-rank test. To examine to what extent the possible predictive factors (residual symptoms, administration of antidepressants and bipolar subtypes) had an impact on the time-to-recurrence curve for the two groups, hazard ratios (HR) and the corresponding 95% confidence intervals (CI) were obtained using a Cox proportional hazard regression model. A p value of less than 0.05 was considered to be statistically significant.

## Results

### Patient disposition and characteristics

**Figure 1:**
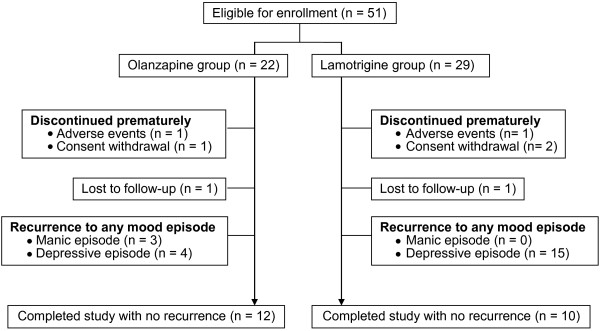
Patient disposition during the study.

Fifty-one patients initially entered this study, with 22 patients in the olanzapine group and 29 in the lamotrigine group during the enrolment period (August 2008 to July 2009). Twenty-two patients (7 in the olanzapine group, 15 in the lamotrigine group) discontinued the study due to recurrence of a mood episode. Two patients (1 in the olanzapine group, 1 in the lamotrigine group) dropped out from the study due to adverse events, and 2 patients (1 in the olanzapine group, 1 in the lamotrigine group) were lost to follow up. Three patients (1 in the olanzapine group, 2 in the lamotrigine group) discontinued the trial after withdrawing consent. Except for the recurrence of any mood episode, the rates of discontinuation were similar between the two groups (Figure 1).

The patients’ demographic and disease characteristics are summarized in Table 1. There were no significant differences in mean age, gender distribution and onset age of bipolar disorder between the two groups. In addition, the proportion of the patients with comorbid personality disorder, comorbid alcohol use disorder and other psychiatric or medical disorders was comparable between the two groups. Furthermore, there were no significant between-group differences in the duration of euthymic state before enrolment, the number of affective episodes in the past year, and the proportion of the participants with subsyndromal residual affective symptoms. However, the proportion of the patients with a diagnosis of bipolar I disorder was significantly greater in the olanzapine group (65.0% vs. 19.2%, χ^2^ = 9.94, p = .002). In addition, there were significant differences between the two groups in the distribution of polarity of the previous episode. Most of the patients in the olanzapine group had experienced manic, hypomanic or mixed previous episodes (n = 13, 65.0%), whereas most of the patients in the lamotrigine group were depressed before enrolment (n = 22, 84.6%, χ^2^ = 11.94, p = .001). In addition, the olanzapine-treated patients also had a higher present YMRS score (6.3 ± 5.0 vs. 3.3 ± 3.7 (mean ± standard deviation), t-test = 2.32, p = .025) but a comparable present HAM-D score to the lamotrigine-treated patients (4.8 ± 1.1 vs. 5.2 ± 1.0, t-test = -1.43, p = .161). In terms of the medications, the mean dose for the patients treated with olanzapine was 10.9 ± 7.4 mg/day (mean ± standard deviation), and the mean dose for those treated with lamotrigine was 86.5 ± 49.6 mg/day. Compared to the olanzapine group, more lamotrigine-treated patients required the administration of concomitant antidepressants (69.2% vs. 30.0%, χ^2^ = 6.97, p = .008).

**Table 1:** Demographic and disease characteristics of the bipolar disorder patients in the euthymic state

	**Olanzapine (n = 20)**	**Lamotrigine (n = 26)**
Age (years), mean (SD)	43.5 (11.8)	38.8 (11.5)
Female, n (%)	11 (55.0)	21 (80.8)
Age of onset, mean (SD)	33.0 (11.7)	27.4 (7.6)
Polarity of first onset		
Manic, Mixed episode or others, n (%)	7 (35.0)	4 (15.4)
Depressive episode, n (%)	13 (65.0)	22 (84.6)
Diagnosis as Bipolar I disorder, n (%)^a^	13 (65.0)*	5 (19.2)*
Polarity of previous episode^b^		
Manic, Mixed episode or others, n (%)	13 (65.0)	4 (15.4)*
Depressive episode, n (%)	7 (35.0)	22 (84.6)*
With residual affective symptoms, n (%)	9 (45)	8 (30.8)
Weeks of euthymic state before enrolment, mean (SD)	13.1 (8.9)	10.8 (5.8)
Number of depressive episodes, past year, mean (SD)	0.3 (0.4)	0.5 (0.6)
Number of manic, mixed episodes or others, past year, mean (SD)	0.3 (0.5)	0.2 (0.5)
Comorbid personality disorder, n (%)	3 (15.0)	9 (34.6)
Comorbid alcohol abuse/dependence, n (%)	4 (20.0)	4 (15.4)
Comorbid other psychiatric disorder (posttraumatic stress disorder, panic disorder) or medical diseases, n (%)	4 (20.0)	1 (3.8)
Dosage (mg/day), mean (SD)	10.9 (7.4)	86.5 (49.6)
Concomitant medications, n (%)		
Antidepressant(s)^c^	6 (30.0)*	18 (69.2)*
Anxiolytic(s)	7 (35.0)	16 (61.5)
Short-acting hypnotics	20 (100)	21 (80.8)
Long acting hypnotics	12 (60.0)	17 (65.4)
Present YMRS score^d^	6.3 (5.0)	3.3 (3.7)*
Present HAM-D score	4.8 (1.1)	5.2 (1.0)

*Abbreviations:YMRS* Young’s Mania Rating Scale, *HAM-D* Hamilton Depression Rating Scale.

*p < .01; ^a^χ^2^ = 9.94, p = .002; ^b^χ^2^ = 11.94, p = .001; ^c^χ^2^ = 6.97, p = .008; ^d^t-test = 2.32, p = .025.

### Recurrent rates of any mood episode

The major outcome of this study is shown in Table 2. Regarding pole-specific recurrence, olanzapine performed statistically better than lamotrigine in preventing the recurrence of depressive episode (χ^2^ = 6.62, p = .010). In the olanzapine group, only 4 patients (20.0%) had recurrence of depression over the study period compared to 15 (57.7%) in the lamotrigine group. However, olanzapine and lamotrigine had similar efficacy in preventing mania recurrence, with 3 patients (15%) in olanzapine group having recurrent manic episodes during the follow-up period and no lamotrigine-treated patients (χ^2^ = 4.17, p = .075, Fisher’s exact test). In addition, there was no significant difference in the proportion of patients who had recurrence of either manic or depressive episodes between the two groups (olanzapine 7 of 20 (35.0%), lamotrigine 15 of 26 (57.7%),χ^2^ = 2.33, p = .127). Concerning the rates of symptomatic recurrence and syndromic recurrence of manic or depressive episodes according to our definition, the two groups were comparable and there were no significant differences between the groups (Table 2). No recurrence of mixed episodes was noted in this study.

**Table 2:** Major outcome of the one-year follow up study, n (%)

	**Olanzapine (n = 20)**	**Lamotrigine (n = 26)**	**p value**
**Recurrence definition**			
Mania	3 (15.0)	0 ( 0)	0.075^a^
Symptomatic recurrence^b^	1 (5.0)		0.435^a^
Syndromic recurrence^c^	2 (10.0)		0.184^a^
Depression	4 (20.0)	15 (57.7)	0.010*
Symptomatic recurrence^b^	3 (15.0)	9 (34.6)	0.133
Syndromic recurrence^c^	1 (5.0)	6 (23.1)	0.119^a^
Any mood episode	7 (35.0)	15 (57.7)	0.127
Symptomatic recurrence^b^	4 (20.0)	9 (34.6)	0.275
Syndromic recurrence^c^	3 (15.0)	6 (23.1)	0.711^a^

*p < .05.

^a^Fisher’s exact test.

^b^Score ≥ 12 on Young’s Mania Rating Scale or ≥ 7 on Hamilton Depression Rating Scale, or if the patient’s condition required an increase in the dosage of olanzapine or lamotrigine.

^c^The patient’s condition met the DSM-IV-TR criteria for manic or depressive episode or the patient had active suicide ideation.

### Time to recurrence of any mood episode

**Figure 2:**
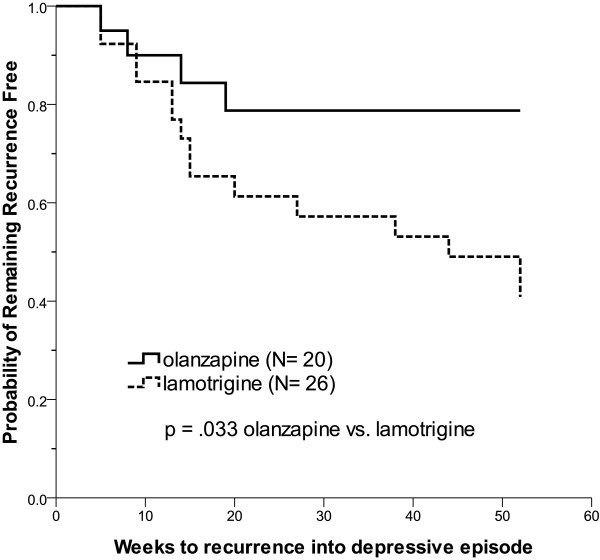
**Time to recurrence into depressive episode: Kaplan-Meier survival curves.** The olanzapine group had a significantly longer time to recurrence into depressive episode than the lamotrigine group (χ^2^ = 4.55, df = 1, p = .033, log-rank test).

**Figure 3:**
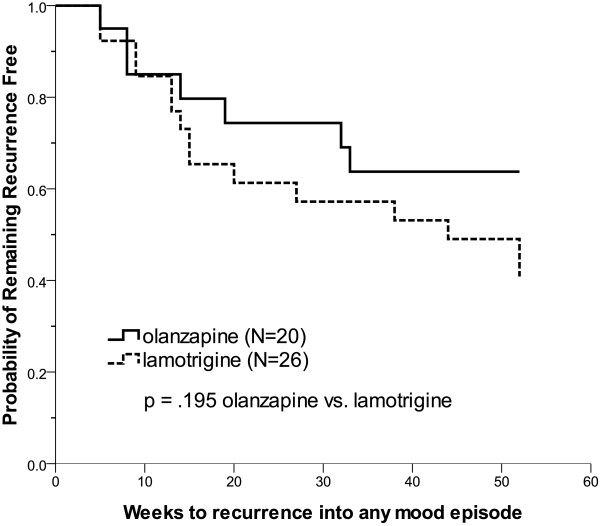
**Time to recurrence into any mood episode: Kaplan-Meier survival curves.** The olanzapine group did not differ from the lamotrigine group in time to recurrence to any mood episode (χ^2^ = 1.68, df = 1, p = .195, log-rank test).

Olanzapine was significantly superior to lamotrigine on the time to recurrence of a depressive episode (χ^2^ = 4.55, df = 1, p = .033, log-rank test, Figure 2). The median time to depression recurrence in the lamotrigine group was 44 weeks; however, there was no sufficient event (4 in 20 patients (20%)) in the olanzapine group to calculate the median time (Table 3). If pole-specificity was not considered, there was no significant difference in the time to recurrence of any mood episode between the olanzapine and lamotrigine groups (χ^2^ = 1.68, df = 1, p = .195, Figure 3). Cox regression analysis with residual affective symptoms, concomitant use of antidepressants and bipolar subtypes as covariates was conducted (Table 4). The patients treated with lamotrigine had a higher risk of recurrence of depression than those in the olanzapine group even with residual affective symptoms (HR 3.8, 95% CI 1.2-11.7, p = .020), concomitant antidepressants (HR 3.9, 95% CI 1.1-12.5, p = .021) or bipolar subtypes (HR 3.8, 95% CI 1.1-13.4, p = .035) being controlled. The risk of the recurrence of depression in the patients with residual symptoms was significantly higher than in those who were asymptomatic (HR 2.6, 95% CI 1.0-6.5, p = .043). Nonetheless, the patients who received antidepressants (HR 0.5, 95% CI 0.2-1.4, p = .209) or had bipolar I disorder (HR 1.5, 95% CI 0.5-4.5, p = .453) did not have a higher risk of depressive episode recurrence. However, we were unable to analyze a gender effect for time-to-recurrence to depression since all of the patients who had depressive recurrence were female.

**Table 3:** Survival data for the bipolar disorder patients in the euthymic state

	**Olanzapine (n = 20)**	**Lamotrigine (n = 26)**	**p value**
Time to recurrence			
Mood episode			
Survival, median (95% CI),	NE	44^a^ (14.2, 73.8)	0.195
No. of events	7	15	
Mania			
Survival, median (95% CI),	NE	NE	
No. of events	3	0	
Depression			
Survival, median (95% CI),	NE	44^a^ (14.2, 73.8)	0.033*
No. of events	4	15	

*Abbreviations:CI* confidence interval, *NE* not evaluable since the treatment groups did not fall below 50% survival.

*p < .05; ^a^Median estimated time to recurrence (weeks).

**Table 4:** Association between hazard ratio for depression recurrence and risk factors of medication and other variables based on a Cox model

	**Crude model**^**a**^		**Multivariable model**^**b**^					
	**HR (95% CI)**	**p Value**	**HR (95% CI)**	**p Value**	**HR (95% CI)**	**p Value**	**HR (95% CI)**	**p value**
Medication								
Olanzapine	Ref.		Ref.		Ref.		Ref.	
Lamotrigine	3.1 (1.0-9.3)	0.045*	3.8 (1.2-11.7)	0.020*	3.9 (1.1-12.5)	0.021*	3.8 (1.1-13.4)	0.035*
Residual affective symptoms								
No symptoms			Ref.					
With symptoms			2.6 (1.0-6.5)	0.043*				
Concomitant medication								
Without antidepressants					Ref.			
With antidepressants					0.5 (0.2-1.4)	0.209		
Bipolar subtypes								
Not Bipolar I							Ref.	
Bipolar I							1.5 (0.5-4.5)	0.453

*Abbreviations: CI* confidence interval, *HR* hazard ratio.

^a^Only medication as the risk factor in the crude model.

^b^Both medication and other variables as the risk factors in the multivariate model.

*p < .05.

### Adverse events

Two patients dropped out from the study due to adverse events. One was treated with lamotrigine and developed a skin rash one week after enrolment, and another patient took olanzapine and discontinued the study in the 47th week of the follow-up period due to obvious weight gain. All of the other participants could tolerate the common side effects of the two drugs such as weight gain and sedation. No patients in the study committed suicide or had severe medication-related complications.

## Discussion

This is the first naturalistic observational study to compare the efficacy of olanzapine and lamotrigine as monotherapy in the prevention of bipolar depression with a one-year follow-up period. The naturalistic design provided a way which is close to clinical practice to observe the direct comparison of these two agents as maintenance treatment in bipolar disorder.

Our results showed that olanzapine had both lower recurrence rate and longer time to recurrence of a depressive episode in the patients already treated with either of these two agents than lamotrigine.

For the patients with bipolar disorder, prophylaxis of recurrence or relapse is the ultimate goal of treatment to mitigate the overall burden of their lives, especially that caused by depressive symptoms [7]. Patients with bipolar disorder suffer from depressive symptoms for the most of time of their lives [6], and therefore medication that can prevent both manic and depressive episodes will be highly beneficial. Nevertheless, among atypical antipsychotics, only quetiapine has been reported to show relatively equivalent efficacy in preventing both manic and depressive episodes [21,22]. Our results demonstrated that olanzapine was effective as prophylaxis treatment for the recurrence of depression. This finding is in contrast to previous reports which showed that olanzapine could prevent depressive episodes only when used in combination with fluoxetine [45] and therefore that olanzapine was only indicated for the prevention of manic episodes as maintenance treatment [46]. However, the effect of olanzapine in preventing depressive episodes was observed in this study. Together with the anti-manic effect of olanzapine, the prevention of depressive episodes suggests the efficacy of maintenance treatment for bipolar disorder with olanzapine in clinical practice.

In addition to the prevention of depressive episodes, we also found that the patients treated with olanzapine had a longer duration to recurrence of depression than those treated with lamotrigine. To control the covariates for the prevention of bipolar recurrence, further analysis of our results showed that the patients who had more residual symptoms but not different bipolar subtypes or concomitant antidepressants had a higher risk for the recurrence of depression. The effect of gender on treatment responsiveness has rarely been discussed in the literature [34,46], and in the current study, all of the participants with recurrence of depressive episodes were female during the follow-up period. Our results are consistent with the findings of a previous investigation conducted by Perlis et al. [11] in that residual symptoms may shorten the duration to recurrence of depression, but male patients are at a lower risk of the recurrence of depression.

The current study has the advantage of using a continuation study design that has rarely been adopted in previous studies on the efficacy of maintenance treatment with olanzapine [46]. Our results demonstrated that continuing the administration of olanzapine after the acute phase in patients with a good response and tolerability to the agent obtained good efficacy in preventing both manic and depressive episodes. To the best of our knowledge, most previous maintenance studies randomly allocated participants to different regimens from the original treatment after remission from the index episode [25,33,45,47,48]. The process of switching drugs or adjusting dosage may induce exacerbation of the illness and subsequent recurrence. A study of continuation design by Tohen et al. [32] comparing the efficacy between olanzapine and divalproex, although it kept the original regimen into and through the maintenance phase, the study design restricted the administration of sedatives for agitation, which may lead to more residual symptoms in the participants in the remission state. Therefore, the recurrence of depressive episodes in that study may have been highly associated with subsyndromal symptoms such like sleep disturbances [49] rather than resulting from the poor efficacy of olanzapine.

It has been reported that lamotrigine is not superior to lithium in the prevention of bipolar depression [39], and also that lithium is not indicated for the prevention of bipolar depression compared to a placebo [50]. Therefore, we speculated that lamotrigine might not be effective in the prevention of bipolar depression, even though two RCTs have indicated its use for maintenance treatment in bipolar depression [37,38]. It is possible that olanzapine was superior to lamotrigine in the prevention of bipolar depression in the current study because lamotrigine does not always show positive efficacy in maintenance treatment [51].

Long-term prophylaxis treatment is usually recommended for the patients with bipolar disorder to prevent the recurrence of any mood episode [52]. Since these patients are likely to have to take medication throughout their lives, the efficacy of the indicated agent should be balanced against its safety and tolerability, which are also associated with non-adherence in patients with bipolar disorder [53]. In recent years, an increasing number of studies have emphasized the metabolic side effects and increased morbidity and mortality related to the use of atypical antipsychotics [41,54], although their role in maintenance treatment has been demonstrated in many RCTs [51]. In contrast, lamotrigine, categorized as a mood stabilizer, has a minimal risk of weight gain [35] while its efficacy in maintenance treatment may only be applicable to bipolar depression [34]. There is therefore a dilemma in clinical practice over how to make a choice between efficacy and safety in long-term prophylaxis treatment of bipolar disorder. Many clinicians may hesitate to prescribe atypical antipsychotics considering their possible adverse effects. However, few clinical trials have been conducted with a parallel comparison study design for the effectiveness of lamotrigine and atypical antipsychotics as maintenance treatment. Our results suggest the better performance of olanzapine both in the prevention of recurrence of mania and depression that should be taken into account when efficacy is the main concern, although future studies are needed to monitor the long-term safety.

There are several limitations to the current study. Initially, the naturalistic design of this study allowed for the prescription of either medication based on clinical judgment according to the patients’ symptoms profile and bipolar subtypes. This may account for the better prevention of depression recurrence of olanzapine, since the patients who were less depressed but more agitated, violent, or aggressive were prescribed olanzapine. In contrast, the patients who had more depressive symptoms were prescribed lamotrigine, and therefore may have been at higher risk of recurrence of depressive episodes. It should also be considered that more patients in the lamotrigine group had bipolar II disorder and they were more likely to have a less favorable course of illness in terms of depressive episodes. In addition, the different symptom profiles between the two groups suggest that the patients maintained with lamotrigine used more antidepressants as concomitant medications to achieve remission from the preceding acute depressive episode. Accordingly, the enriched sample in our study may confine the interpretation of the results only to the patient population that respond to and tolerate these two drugs. Double blind studies with more subjects are needed to confirm our findings.

Additionally, the higher recurrence rate of depression in the patients treated with lamotrigine may be associated with the relatively lower dosage of lamotrigine we administered in this study compared to the 200 mg/day which was recommended in previous RCTs with positive results compared to a placebo [38,55]. Other limitations include the small sample size of both study arms leading to insufficient power, and the short follow-up period so that the results can only be generalised within 52 weeks. An additional limitation was that we did not measure the adverse events resulting from the long-term administration of the two agents quantitatively and in detail. Therefore, we had difficulty in assessing the impact of complications such as metabolic side effects or other physical discomfort on the participants after receiving the prophylaxis treatment.

## Conclusions

We found that olanzapine was more effective than lamotrigine in the prevention of the recurrence of bipolar depression, with a naturalistic study design close to a clinical setting. Further randomized studies comparing these two agents with a larger sample size in the maintenance treatment of bipolar disorder are required to validate the findings of this study.

## Competing interests

The authors declare no conflict of interest of this article.

## Authors’ contributions

CB designed and carried out the study, participated in drafting the manuscript. PY undertook the statistical analyses, interpreted the data, and wrote the first draft of the current manuscript. MS helped interpret the data, and revised the current manuscript critically. MC and EL carried out the data collection and preliminary statistical analyses. All authors read and approved the final manuscript.

## Pre-publication history

The pre-publication history for this paper can be accessed here:

http://www.biomedcentral.com/1471-244X/14/145/prepub
